# Relevance of Cellular Redox Homeostasis for Vital Functions of Human Dental Pulp Cells

**DOI:** 10.3390/antiox11010023

**Published:** 2021-12-23

**Authors:** Marialucia Gallorini, Matthias Widbiller, Carola Bolay, Simone Carradori, Wolfgang Buchalla, Amelia Cataldi, Helmut Schweikl

**Affiliations:** 1 Department of Pharmacy, University “G. d’Annunzio” Chieti-Pescara, Via dei Vestini 31, 66100 Chieti, Italy; cataldi@unich.it; 2 Department of Conservative Dentistry and Periodontology, University Hospital Regensburg, 93042 Regensburg, Germany; matthias.widbiller@ukr.de (M.W.); carola.bolay@ukr.de (C.B.); wolfgang.buchalla@ukr.de (W.B.); helmut.schweikl@ukr.de (H.S.)

**Keywords:** IL-6, oxidative stress, inflammation, mineralization, dental pulp, fisetin, LPS, immunophenotype, BMP2, ALP

## Abstract

Odontogenic MSCs are vulnerable to LPS-triggered bacterial infections, and they respond by secreting inflammatory mediators, such as IL-6, and with mineralization. Since both processes might be prone to a disturbance of the redox homeostasis, the oxidative stress influence on vital functions of human dental pulp cells (HPCs) was investigated. With these aims, a model of LPS-stimulated primary HPCs was established, and anti- and pro-oxidant substances were administered up to 21 days to measure inflammation and mineralization parameters. LPS-stimulated HPCs retained mineralization potential, which was decreased with the antioxidants NAC and fisetin and the pro-oxidant BSO. The expression of surface markers related to odontogenic commitment was influenced accordingly but counteracted by the enhanced expression of BMP2 and ALP at the transcriptional level. LPS triggers an early IL-6 production in non-odontogenic conditions, while it can be measured only after 15 days in the presence of the differentiation medium. The present study shows that HPCs functions causally depend on a tightly regulated cellular redox balance. Our data demonstrate a redox control of pulp MSC odontogenic commitment along with a potential association between an IL-6 late secretion and mineralization. These findings lay the groundwork for investigations on the molecular role of IL-6 in dental hard tissue metabolism.

## 1. Introduction

Interleukin-6 (IL-6) acts as a pleiotropic cytokine, due to its multiple roles in several tissues and organs. Typically, IL-6 has been investigated in immune cells, coordinating fundamental processes related to adaptive immune responses and being involved in acute phase reactions against pathogens and chronic inflammation. However, since it is secreted by many other cell types, IL-6 has been reported to play a role in other physiological processes [1]. Recently, the multifunctional role of IL-6 has been investigated in regenerative processes, and it was observed that mesenchymal stem cells (MSCs) release extremely high levels of IL-6 compared to other cytokines. However, there is still little known about the precise IL-6 function in the homeostasis of MSCs and the role of these cells in immunoregulation under reparative processes [2]. Xie and colleagues [3] reported that MSCs from the bone marrow both secrete and respond to IL-6 and these high expression levels of IL-6 and its receptor promote MSCs osteogenic differentiation. In a clinical situation of fracture repair, the osteogenic commitment of MSCs can be complicated by the elevated levels of interleukins which specifically amplify the inflammatory environment [4]. It has been shown that a rise in pro-inflammatory mediators such as interleukin (IL)-1β inhibits bone morphogenic protein 2 (BMP2)-induced osteogenic differentiation of MSCs [5]. However, IL-6 seems to function differently because a synergetic effect with BMP-2 to enhance bone regeneration was shown in an animal model [6].

A subpopulation of multipotent undifferentiated MSCs of neural crest origins also resides within the dental pulp of human teeth capable of undergoing odontogenic, adipogenic and neurogenic differentiation [7]. Moreover, cells from the dental pulp express surface markers typically related to a mesenchymal origin [8] and they own a peculiar genetic profile characterized by markers related to osteogenesis, such as BMP-2 and alkaline phosphatase (ALP) during standard procedures for odontogenic differentiation in vitro [9]. The dental pulp is thus the residence of odontogenic MSCs, which are essential for the process of tooth development and injury repair through the secretion of reparative dentin. Nonetheless, the regenerative abilities of these cells are easily affected by the environmental conditions of their niche. As a matter of fact, dental pulp is vulnerable to trauma and bacterial infections, and the secretion of cytokines triggered by bacterial products such as lipopolysaccharide (LPS) may influence the mineralization potential of odontogenic pulp cells [10,11].

Therefore, investigating the close link between inflammation and the induction of regenerative processes in dental pulp is of fundamental importance to disclose whether this interplay may influence dentin and pulp regeneration. In a clinical situation, after injuries or bacterial infiltrations, cytokines are released from odontoblasts and probably mesenchymal pulp cells to finally initiate dental pulp inflammation as a mechanism of defense. These adaptive cell responses are related to oxidative and nitrosative stress, caused by reactive oxygen or nitrogen species (ROS or RNS, respectively). Dysregulated high levels of ROS or RNS may lead to apoptosis and cell cycle arrest in MSCs [12,13]. In parallel, several recent studies reported on the importance of a redox control of MSC differentiation [14]. Our group has previously observed molecular pathways underlying the intracellular balance between redox homeostasis and extracellular matrix mineralization of human pulp cells (HPCs), emphasizing IL-6 as one of the major players involved [15,16]. Furthermore, a redox control through the ROS-activated MAPKs (mitogen-activated protein kinases) signaling has been highlighted under the osteogenic commitment of HPCs in vitro [17].

On this basis, the present study aims to demonstrate the relevance of redox homeostasis for vital HPC functions such as immune response and differentiation. It is assumed that redox control is crucial under the odontogenic commitment of mesenchymal pulp cells and that IL-6 secretion could be a link in the interplay between oxidative stress and mineralization pathways. To this end, primary HPCs were grown in normal and odontogenic media, stimulated with a low concentration of LPS capable of creating a redox homeostasis perturbance, and exposed to anti- or pro-oxidant substances up to 21 days. As an inhibitor of glutathione (GSH) synthesis, a major non-enzymatic antioxidant, buthionine sulfoximine (BSO) was used to further enhance oxidative stress. On the contrary, N-acetylcysteine (NAC) as an antioxidant by itself and as a substrate in GSH synthesis keeps GSH at high levels [18]. In addition, the dietary flavonoid fisetin was included in this study. It has shown strong anti-inflammatory and antioxidant effects in cell cultures and in animal models relevant to human diseases [19]. Here, the expression of distinctive MSC-associated surface markers was analyzed and was shown to be dependent on redox homeostasis. In addition to the analysis of cell viability and the formation of oxidative stress, relevant parameters indicating mineralization have been analyzed. In parallel, a time-course of IL-6 secretion has been established to provide evidence for the simultaneous activation of immune responses.

## 2. Materials and Methods

### 2.1. Chemicals

Minimum essential medium (MEM) α (alpha modification) and fetal bovine serum (FBS) were purchased from Gibco (Thermo Fisher Scientific, Waltham, MA, USA). Lipopolysaccharide (LPS; *E. coli*, serotype 055:B5), L-buthionine sulfoximine (BSO; CAS-No. 83730-53-4), N-acetylcysteine (NAC; CAS-No. 616-91-1), 3-(4,5-dimethylthiazol-2-yl)-2,5-diphenyltetrazolium bromide (MTT), Alizarin red S (A5533), dexamethasone (D4902), ß-glycerophosphate (G9422), penicillin/streptomycin (P0781) came from Sigma Aldrich GmbH (Taufkirchen, Germany). Fisetin (S2298; CAS-No. 345909-34-4) was obtained from Selleck Chemicals (Biozol, Munich, Germany), 2′,7′-dichlorodihydrofluorescin diacetate (DCFH2-DA; CAS-No. 4091-99-0) came from MoBiTec (Göttingen, Germany), ascorbic acid was purchased from Merck (Darmstadt, Germany), and an interleukin 6 (IL-6) ELISA kit was obtained from BD Biosciences (San Diego, CA, USA). Anti-human CD90-FITC (clone 5E10) came from BD Biosciences (San Diego, CA, USA), a monoclonal human 5’-nucleotidase/CD73-PE-conjugated antibody (clone 606112, FAB5795P) was provided by R & D Systems (Minneapolis, MN, USA), monoclonal anti-human CD105 (Endoglin)/R-PE (326-050) was purchased from Ancell (Biomol GmbH, Hamburg, Germany), CD140a-FITC mouse monoclonal (130-115-336) was supplied by Miltenyi Biotec GmbH (Bergisch Gladbach, Germany), and CD45-FITC monoclonal antibody (HI30) (11-0459-42), mouse IgG1 kappa isotype control (FITC) (11-4714-42), mouse IgG1 isotype control (PE) (MA1-10415) and Stem Pro™ Accutase™ were purchased from Thermo Fisher Scientific (Waltham, MA, USA). All other chemicals used in the present study were of at least analytical grade.

### 2.2. Cell Culture

Cell cultures were established from a human third molar after informed consent and approval by the Ethics Committee of the Faculty of Medicine at the University of Regensburg (reference 16-101-0022, date of approval 16 March 2016). Human primary dental pulp cells (HPCs) from the root section of the pulp explant (HPC-10 m) were routinely cultivated in MEM α supplemented with 2.2 g/L of sodium bicarbonate (NaHCO_3_), 10% fetal bovine serum (FBS), 500 U/mL of penicillin, and 0.5 mg/mL of streptomycin in a humidified atmosphere containing 5% CO_2_ at 37 °C as described earlier [20]. Cells were used up to the sixth passage after thawing.

### 2.3. MTT Assay

Cells from routine culture were seeded (7 × 10^3^/well; 96-well plates) in 200 µL of routine culture medium (complete MEM α, M1) and incubated for 48 h at 37 °C and 5% CO_2_. Next, cell cultures were exposed to LPS (0–50 µg/mL), NAC (0–20 mM), BSO (0–200 µM), or to fisetin (0–200 µM) for 24 h or 72 h in the routine culture medium M1, M1 supplemented with 10 nM dexamethasone and 10 mM β-glycerophosphate (M2), or M1 supplemented with 10 nM dexamethasone, 10 mM β-glycerophosphate and 50 µg/mL ascorbic acid (M3). Before administering NAC to cell cultures, solutions were conditioned at 37 °C and 5% CO_2_ to allow for pH neutralization. After that, the exposure media were replaced by 200 µL/well of a solution of MTT (0.5 mg/mL) in M1, and cell cultures were incubated at 37 °C for 3 h in a humidified 5% CO_2_-atmosphere. Then, the MTT solution was removed and replaced with 200 µL/well of DMSO (dimethyl sulfoxide) and gently swirled for 10 min. The optical density in each well was immediately measured using a spectrophotometer (Infinite F200, TECAN, Maennedorf, Zurich, Switzerland) at a wavelength of 540 nm. Each experiment was performed two times in duplicates per experimental condition (n = 4). The optical density readings obtained from treated cell cultures were summarized and normalized to untreated cell cultures (100%). The influence of the various supplements added to the routine culture medium (M2, M3) on cell viability was analyzed accordingly and is shown in the Supplementary Materials (M1 = 100%).

### 2.4. Detection of Reactive Oxygen Species (ROS)

Cells from routine culture were seeded (5 × 10^4^/well) in 2 mL of M1 in 6-well plates and allowed to adhere for 48 h. Cell cultures to be treated with BSO were pre-incubated 24 h after seeding with 50 µM BSO for 18 h. Then, cell cultures were exposed to BSO (50 µM), NAC (2 mM or 5 mM) or fisetin (5 µM, 25 µM or 50 µM) both in the presence or absence of LPS (0.1 µg/mL).

The exposure of cultures was stopped after 3 h or 24 h by discarding the exposure media, and the intracellular production of ROS was measured by flow cytometry after staining the cells with the fluorescent dyes 2′,7′-dichlorodihydrofluorescin diacetate (DCFH2-DA). Favorable properties of this dye for the investigation of oxidative stress have been outlined previously [21]. Cell cultures were incubated with 10 µM DCFH2-DA in culture medium 30 min prior to harvesting in phosphate-buffered saline (PBS)/5 mM ethylenediaminetetraacetic acid (EDTA). Then, the cells were collected by centrifugation and the cell pellet was resuspended in 200 μL calcium and magnesium-free PBS. 2′,7′-dichlorofluorescein (DCF) fluorescence was measured in the FL-1 channel (488/515-545) using a BD FACSCanto (Becton Dickinson) flow cytometer. Mean fluorescence intensities (MFIs) were established using histogram statistics and FACSDiva^TM^ 5.0.2 (Becton Dickinson) software. Individual fluorescence intensities measured in treated cell cultures were related to fluorescence quantified in unstained control cultures.

### 2.5. Immunophenotyping In Vitro by Flow Cytometry

The expression of surface markers in primary pulp cell cultures was analyzed by flow cytometry. Cells from routine cultures were seeded (5 × 10^4^/well) in 3 mL of the M1 medium in 6-well plates and then they were let to adhere for 48 h. Cell cultures to be treated with BSO were pre-incubated 24 h after seeding with 50 µM BSO for 18 h in M1 culture medium. Cell cultures were then treated with BSO, NAC or fisetin in the presence or absence of LPS for 24 h or 3 days in the M1 or the M2 medium. After that, cells were harvested with 3 mL of Stem Pro™ Accutase™, collected by centrifugation in the cold, and washed once with FACS buffer made by 10 mM 4-(2-hydroxyethyl)-1-piperazineethanesulfonic acid (HEPES) buffer at pH 7.4, 140 mM sodium chloride (NaCl) and 2.5 mM calcium chloride (CaCl_2_. Cells were incubated with fluorochrome-conjugated antibodies (1:50 dilutions) in 50 μL of FACS buffer for 15 min in the dark. Cells were stained separately in each single screening tube with cluster of differentiation (CD)90-FITC, CD73-PE, CD105-PE, CD140a-FITC or isotype controls (FITC or PE). Then, the excess of antibodies was removed by adding fresh FACS buffer and centrifugation. After that, 20,000 events were run in a BD FACSCanto flow cytometer (BD Biosciences). Relative fluorescence emissions of gated cells by forward and side scatter properties (FSC/SSC) were analyzed with FACSDiva^TM^ 5.0.2 (BD Bioscience) and expressed as mean fluorescence intensities (MFIs). The MFI ratio was calculated by dividing the fluorescence of the sample by the fluorescence of the related isotype control. Individual values obtained from independent experiments were summarized as means and standard deviations.

### 2.6. Odontogenic Differentiation In Vitro

Cells from routine culture were seeded (2 × 10^4^/well) in 0.5 mL of M1 in 48-well plates and then let to adhere for 48 h. Cell cultures to be treated with BSO were pre-incubated after 24 h from the seeding with 50 µM BSO for 18 h in M1 medium. Then, the cultures were exposed to BSO, NAC or fisetin in the presence or absence of LPS in M1 or M2 medium. Fresh exposure media were provided every 72 h. The exposure of cultures was stopped after 1, 3, 7, 15 (data not shown), or 21 days by discarding the exposure media. Then, cell cultures were washed with PBS and fixed with 10% paraformaldehyde for 15 min at room temperature. Calcium precipitation was detected after Alizarin staining of cell cultures following an established procedure [20].

### 2.7. Semi-Quantitative Real-Time PCR

Semiquantitative real-time PCR (TaqMan, Thermo Fisher Scientific, Waltham, MA, USA) was performed to analyze the expression of BMP-2 (Hs00154192_m1) and ALP (Hs01029144_m1) with RPS18 (Hs01375212_g1), 18S (Hs99999901_s1) and ACTB (Hs01060665_g1) as housekeeping genes. Cells from routine culture were seeded (2 × 10^4^/well) in 0.5 mL of M1 in 48-well plates in triplicate and then they were let to adhere for 48 h. Cell cultures to be treated with BSO were pre-incubated after 24 h from the seeding with 50 µM BSO for 18 h in M1 medium. Then, the cultures were exposed to BSO, NAC or fisetin in the presence or absence of LPS in M1 or M2 medium. Fresh exposure media were provided every 72 h. The exposure of cultures was stopped after 1 (data not shown), 3, 7, 15 (data not shown), or 21 days by discarding the exposure media, and cell pellets were obtained by harvesting cells after incubation with Accutase™ and by centrifugation. RNA was isolated from cell pellets by RNeasy Mini Kit (Qiagen, Hilden, Germany), quantified spectrophotometrically, and 200 ng was transcribed into first strand cDNA in a volume of 20 µL (Omniscript RT Kit, Qiagen, Hilden, Germany). Real-time PCR was performed in a total volume of 20 µL containing 1 µL of TaqMan Assay, 10 µL TaqMan Fast Advanced Master Mix, 7 µL H_2_O and 2 µL cDNA (1:8 diluted). After denaturation at 95 °C (20 s), 40 sequential cycles were performed with denaturing at 95 °C (1 s) and annealing/elongation at 60 °C (20 s) in a QuantStudio 3 Real-Time PCR System (Thermo Fisher Scientific, Waltham, MA, USA). Delta Ct-values of BMP-2 and ALP were calculated by normalization on RPS18, 18 s and ACTB in average, and fold changes were presented relative to M1 or M2 at day 3 (Delta-Delta Ct-method). Results were summarized from two independent experiments performed in duplicates (n = 4).

### 2.8. Determination of IL-6

Exposure media collected every 72 h from 48-well plates for semi-quantitative real-time PCR were used for the analysis of interleukin-6 (IL-6). IL-6 secretion was quantified using a standard ELISA kit containing biotinylated anti-human monoclonal antibodies following the manufacturer’s instructions (BD Biosciences). The absorbance was read at 450 nm by means of a spectrophotometer (Infinite F200, TECAN, Mainz, Germany), optical densities were collected (Magellan software; version 6.2) and IL-6 concentrations were calculated fitting the obtained optical densities in a standard curve ranging from 4.7 to 300 pg/mL. Detectable amounts of IL-6 were summarized from duplicate measurements in independent experiments.

### 2.9. Statistical Analysis

Statistics were performed using one-way analysis of variance (ANOVA) followed by Tukey’s multiple comparison test by means of the Prism 5.0 software (GraphPad, San Diego, CA, USA). Results are presented as mean values ± standard deviations. Values of *p* ≤ 0.05 were considered statistically significant.

## 3. Results

### 3.1. Cell Metabolic Activity (MTT Assay)

**Figure 1:**
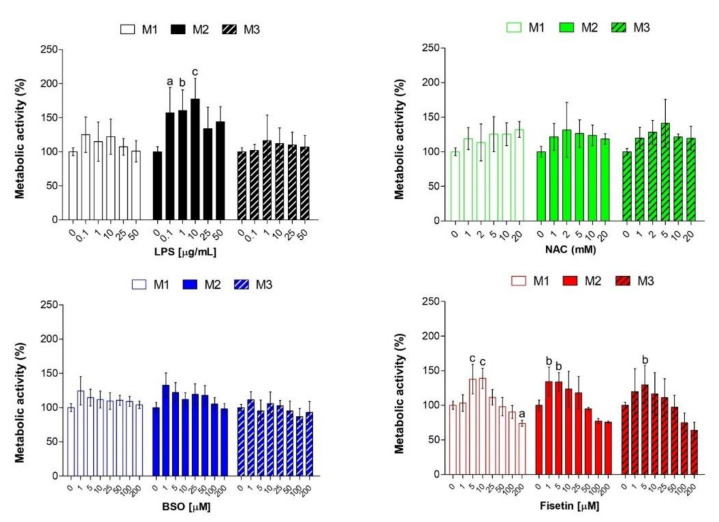
Cell metabolic activity in human pulp cells. Cell cultures were exposed to LPS, NAC, BSO or fisetin for 24 h. Optical density readings in untreated cultures (0 µM) were set to 100%. Bars show mean values ± standard deviations summarized from individual values in independent experiments (n = 4). M1 = complete growth medium; M2 = complete growth medium with dexamethasone and β-glycerophosphate; M3 = complete growth medium with dexamethasone, β-glycerophosphate and ascorbic acid. Lower case letters indicate significant differences between untreated cell cultures (0 µM) and treated samples: a (*p* < 0.01), b (*p* < 0.001) and c (*p* < 0.0001).

Initially, pulp cells were exposed to increasing concentrations of all substances tested (LPS, NAC, BSO and fisetin) in the presence of the M1, M2 and M3 media for 24 h (Figure 1) and 72 h (Figure S2) and cell metabolic activity was assessed by the MTT assay as reported elsewhere [22]. The influence of the three different growth solutions on routinely cultured pulp cells is not significantly relevant, as shown in Figure S1. Increasing concentrations of LPS are furthermore slightly effective after 24 h when HPCs are stimulated in the presence of M1 and M3 (Figure 1). Notably, cell viability increases about 1.5-fold when cells are stimulated with LPS in M2, mainly in the 0.1–10 µg/mL range. The same pattern is registered after 72 h but to a lesser extent. As for NAC exposure, percentages of HPC metabolic activity are only slightly increased with respect to untreated cultures under all the experimental conditions. However, after 72 h a dose-dependent decrease in metabolic activity can be registered, disclosing cytotoxicity of NAC in the 10–20 mM concentration range independently from the growth medium. Surprisingly, the administration of BSO is ineffective in HPC over the chosen concentration range (0–200 µM), and cell metabolic activity is even slightly but not significantly increased in the presence of 1–50 µM BSO. In parallel, percentages of metabolically active HPCs remain at constant levels of untreated cells after a 72-h exposure to BSO. Finally, a bell curve can be observed when HPCs are exposed to fisetin for 24 h. While the percentages of cell metabolic activity significantly increased in the lowest concentration range (1–25 µM), a dose-dependent decrease in HPC metabolic activity can be registered in the 50–200 µM range independent from media. The percentage of viable cell numbers dramatically lowers after a 72-h exposure, to 25–200 µM fisetin (Figure S2). Since a modulation in cell viability can be observed already at LPS 0.1 µg/mL, this concentration was chosen to stimulate HPCs for further experiments. Next, NAC 2 and 5 mM were furthermore used due to the absence of cytotoxicity both after 24 h and 72 h. As for BSO, the concentration of 50 µM, which was plausibly able to interfere with cell redox homeostasis, was picked in line with our previous results [18]. Finally, since cell metabolic activity is clearly influenced by fisetin in a dose-dependent manner, concentrations of 5, 25 and 50 µM were chosen for further experiments, excluding the highest concentration range due to the cytotoxicity registered.

### 3.2. Generation of Reactive Oxygen Species

**Figure 2:**
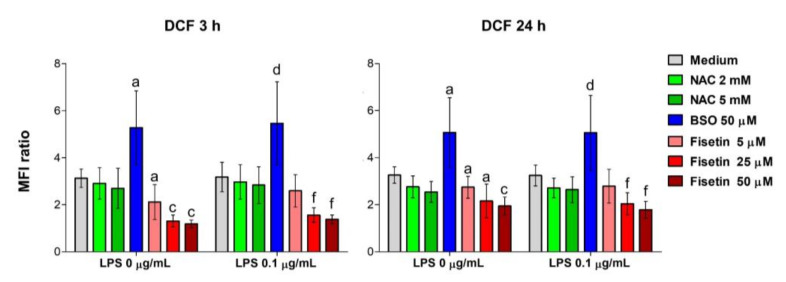
Generation of reactive oxygen species in human pulp cells. Cell cultures were treated under various experimental conditions in complete growth medium (M1) for 3 h or 24 h. The bar graphs represent the mean fluorescence intensity (MFI) ratio related to the formation of reactive oxygen species detected after staining with DCF. Values found with unstained sample are set to 1 (not shown). Bars show mean values ± standard deviations summarized from individual values in independent experiments (n = 6). Significant differences between 0 µM (=M1) and treated samples: a (*p* < 0.01) and c (*p* < 0.0001) between 0 µM and treated samples without LPS (0 µg/mL); d (*p* < 0.01), and f (*p* < 0.0001) between LPS alone and treated samples in the presence of LPS (0.1 µg/mL). BSO increases the amounts of reactive oxygen species (ROS) while the antioxidants NAC and fisetin reduce ROS levels independently from LPS.

To determine whether ROS generation is modified, HPCs were exposed to NAC, BSO and fisetin with or without 0.1 µg/mL LPS for 3 h or 24 h (Figure 2). Cells were exposed in M1 medium only to verify the effect of the single substances without any interference related to additives in M2 and M3. In general, the pattern registered after 3 h and 24 h reveals that the levels of ROS produced are independent of LPS or exposure periods. Physiologically, the amount of ROS generated in untreated pulp cells is 3.1-fold (MFI) greater than the amount in the unstained sample under all experimental conditions. The presence of 2- or 5-mM NAC slightly reduces DCF fluorescence. As expected, BSO exposure considerably amplifies the amounts of ROS by about five-fold compared to untreated controls. Finally, a significant dose-dependent decrease in ROS can be registered in the presence of fisetin with or without LPS already after 3 h and at the lowest concentration of 5 µM (two-fold). After that, the amount of ROS is even more decreased, being comparable to one of the unstained samples (around 1.1 with 50 µM fisetin).

### 3.3. Immunophenotype

**Figure 3:**
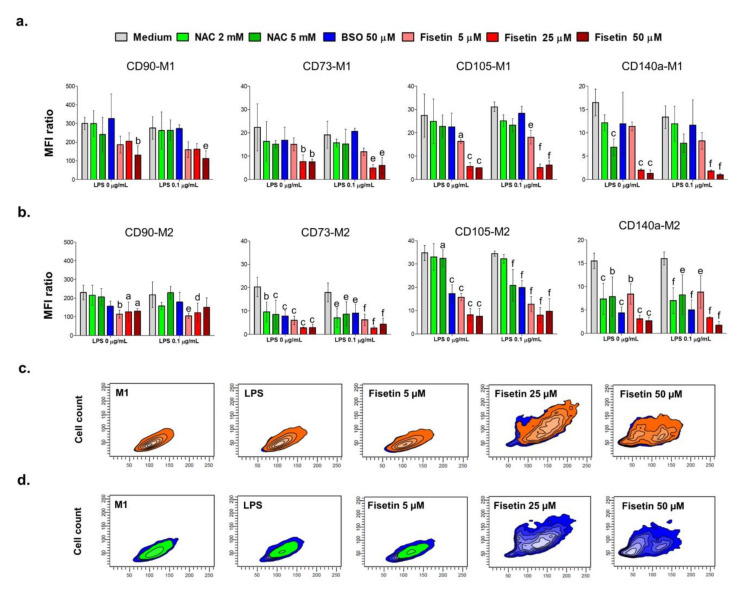
Immunophenotypic profile of human pulp cells. Cell cultures were treated under various experimental conditions for 3 days. Mean fluorescence intensity ratios (MFI ratio) for surface markers CD90, CD73, CD105, and CD140a with M1 (**a**) and M2 (**b**) were obtained as described in the Materials and Methods section. Bars represent the MFI of the sample divided by the fluorescence intensity of related isotype controls as mean values ± standard deviations summarized from individual samples in two independent experiments (n = 4). M1 = complete growth medium; M2 = complete growth medium with dexamethasone and β-glycerophosphate. a = *p* < 0.01, b = *p* < 0.001 and c = *p* < 0.0001 between 0 µM and treated samples without LPS (0 µg/mL); d = *p* < 0.01, e = *p* < 0.001 and f = *p* < 0.0001 between LPS alone and treated samples in the presence of LPS (0.1 µg/mL). Forward scatter/Side scatter (FSC/SSC) density dot plots from one representative experiment represent the cell population CD73-positive in orange (**c**), CD140a positive in green (**d**) and unstained in blue. The immunophenotypic profile of human pulp cells is weakly influenced by LPS and strongly modulated by fisetin.

The MSC-related surface markers CD90, CD73, CD105 and CD140a were quantified in HPCs in the various experimental conditions after 1–3 days to evaluate whether a modulation occurs in the presence of substances capable of interfering with cell redox homeostasis (Figure 3). Some overall observations can be listed prior to analyzing the results in greater detail. Firstly, it seems that the LPS stimulation does not have an influence on the modulation of CD markers (Figure 3a,b). Secondly, the expression of CD markers is weakly affected after 1 day of exposure (Figure S3) to NAC or BSO, whereas fisetin demonstrates its effectiveness also after short exposure times. Thirdly, the CD marker profile appears to be differentially modulated depending on the growth medium (M1 or M2) after 3 days, mainly in the presence of NAC or BSO, whereas the effect of fisetin is not influenced by the media. In details, the expression of CD90 significantly decreases in the presence of M2 under all experimental conditions compared to samples in the presence of M1. For instance, the MFI ratio is assessed at 300.5 with M1 and at 230.3 with M2 for the untreated control (medium). As for NAC or BSO, a slight modulation of CD90 can be registered in all the experimental conditions, but to a not significant extent. Contrariwise, CD90 expression levels lower in the presence of fisetin, independently from the medium, the presence of LPS and the concentration. Next, the amount of CD73 on the surface membrane of HPCs is not significantly affected neither by NAC nor by BSO in the presence of M1 compared to untreated controls (MFI ratio = 22.36), while fisetin lowers CD73 levels in a dose-dependent manner independently from LPS (MFI ratio around 7 for 50 µM fisetin). In contrast, all substances tested strongly decrease CD73 expression in the presence of M2, with fisetin again showing to be the most effective. It is worth noting that fisetin does not influence the positivity of HPCs for CD73 compared to M1 and LPS, as shown by density dot plots (Figure 3c). After that, the expression of the endoglin CD105 was analyzed, revealing that NAC exposure is slightly effective on the modulation of this marker in all the experimental conditions, although a decrease could be noticed. The only exception is represented by a decrease in LPS-stimulated HPCs in the presence of 5 mM NAC (MFI ratio = 8.63) compared to LPS alone (MFI ratio = 11.27). An influence on CD105 expression in the presence of M1 was not detected with 50 µM BSO, whereas it is significantly effective in the presence of M2 independently from the LPS stimulation. As for previously described markers, fisetin dramatically lowers the amount of CD105 in a dose-dependent manner in all the experimental conditions. Finally, levels of CD140a were quantified. All substances administered decrease CD140a expression, mainly in the presence of M2, independently from LPS stimulation. Once again, fisetin is the most effective substance in lowering CD140a expression. Contrary to what has been reported for CD73, the CD140a cell positivity is hardly detectable for HPCs treated with 25 and 50 µM fisetin compared to untreated cells, LPS alone and 5 µM fisetin (Figure 3d).

### 3.4. Mineralized Matrix Deposition and Microscopic Observation

**Figure 4:**
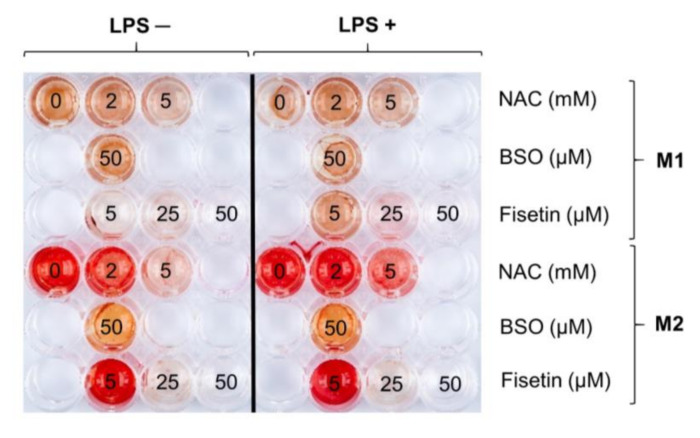
Mineralization occurrence in human pulp cells after 21 days in the various experimental conditions. Images of cell cultures in situ after staining with Alizarin red. M1 = complete growth medium; M2 = complete growth medium with dexamethasone and β-glycerophosphate. Numbers indicate concentrations of NAC, BSO or fisetin (in mM or µM). Mineralization in human pulp cells is downregulated in the presence of the prooxidant BSO and the antioxidants NAC and fisetin.

After having quantified the expression of CD markers related to HPC odontogenic potential after short times of exposure, their ability to mineralize the extracellular matrix was analyzed up to 21 days. After 21 days of exposure the M2 medium enhances the deposition of calcium crystals from HPCs, and the phenomenon is independent from LPS stimulation (Figure 4). A dose-dependent decrease in the mineralization capacity occurs when HPCs are cultivated in the presence of increasing concentration of NAC independently from the medium. Interestingly, cells retain their mineralization capability when stimulated with LPS. In the presence of 50 µM BSO and M1, the intensity of the staining is comparable to that of untreated cultures, also when cells are LPS-stimulated. Contrariwise, when cells are cultivated in the presence of the M2 medium, the BSO lowers the mineralization capacity of HPCs. Finally, a dose-dependent decrease in the Alizarin red staining intensity can be detected in the presence of fisetin. In the presence of the M1 medium without LPS, a red staining is hardly detectable even with the lowest concentration of fisetin (5 µM). When HPCs are stimulated with LPS in the presence of 5 µM fisetin, the staining intensity is comparable to that of cells in the presence of LPS alone, whereas 25 and 50 µM decrease HPCs mineralization capacity as in HPCs without LPS. In parallel, when HPCs are cultivated in the M2 medium and 5 µM fisetin is administered, cells are red stained as the ones of the untreated control, independently from the LPS stimulation. Fisetin induces a dose-dependent decrease in the mineralization capacity, and a red staining is virtually absent in cultures treated with 50 µM fisetin. Since fisetin is the only substance which shows this dramatical reduction or even a total lack of alizarin red staining intensity in parallel with a significant decrease in cell viability (Figure 1), cultures underwent a microscopic analysis to investigate whether the morphological changes in the cell monolayer or cell density may influence calcium deposition (Figure S4). After 3 days of culture in the presence of the M1 medium, untreated HPCs (A) regularly grow as a monolayer and show a typical spindle-shaped fibroblast-like morphology. The presence of LPS (F) seems not to affect HPC proliferation and morphology. When increasing concentrations of fisetin are added to M1 (B, C and D), the cell density dramatically decreases. Moreover, a change in cell morphology can be observed as HPCs are extremely elongated and hexagonal with longer protracted processes. In addition, secretory vesicles can be observed at the highest concentration of 50 µM (E and L).

### 3.5. Expression of Genes Related to Mineralization

**Figure 5:**
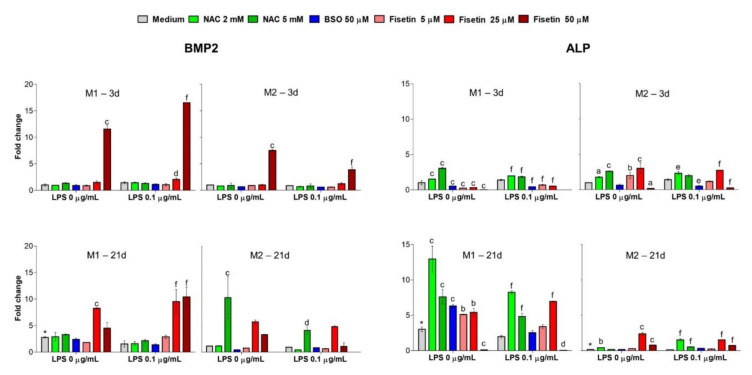
Gene expression of bone morphogenic protein 2 (BMP2) and alkaline phosphatase (ALP) in the various experimental conditions. Bars represent delta CT-values of gene expression as mean values ± standard deviations normalized to RPS18 expression. Data presented are summarized from three independent experiments (n = 4). M1 = complete growth medium; M2 = complete growth medium with dexamethasone and β-glycerophosphate. a = *p* < 0.01, b = *p* < 0.001 and c = *p* < 0.0001 between 0 µM and treated samples without LPS (0 µg/mL); d = *p* < 0.01, e = *p* < 0.001 and f = *p* < 0.0001 between LPS alone and treated samples in the presence of LPS (0.1 µg/mL); * = *p* < 0.01 between M1 or M2 after 3 days and M1 or M2 after 21 days. The expression of BMP2 and ALP depends on the cellular redox homeostasis, which is modified by the pro-oxidant BSO and the antioxidants NAC and fisetin.

After having observed the pulp cell capacity to mineralize the ECM, the expression of molecules (BMP2 and ALP) related to odontogenesis was detected at the gene level after 3 and 21 days in the presence of the M1 and M2 media (Figure 5). After 3 days, both NAC and BSO do not affect BMP2 levels, in the presence of neither M1 or M2. Surprisingly, 50 µM fisetin significantly upregulates BMP2 expression both in LPS-stimulated and -unstimulated cultures after 3 days independently from the medium. The absolute highest increase related to BMP2 can be registered for LPS-stimulated HPCs in the M1 medium supplemented with 50 µM fisetin (16.5-fold). As expected, after 21 days of culture in M1, BMP2 expression is slightly enhanced in untreated cultures (2.8-fold) compared to M1 after 3 days. Again, fisetin dramatically upregulates the BMP2 gene, starting from a 25 µM concentration (8.2-fold without LPS and 9.5-fold with LPS). LPS stimulation enhances the expression of BMP2 with 50 µM fisetin (10.4-fold) compared to unstimulated cells (4.5-fold). When HPCs were grown in the presence of the M2 medium for 21 days, 5 mM NAC significantly enhances BMP2 expression with and without LPS (4.1- and 10.2-fold, respectively). Gene expression of ALP was quantified under the same experimental conditions. After 3 days in the presence of the M1 medium, increasing concentrations of NAC upregulate ALP independently from LPS. On the other hand, when HPCs were cultivated in the presence of the M2 medium, also 5 and 25 µM fisetin almost triplicate ALP levels of expression, whereas ALP transcripts are hardly detectable with 50 µM fisetin. After 21 days of cultivation in M1, the expression of ALP in untreated cells increases almost 3-fold compared to cells cultured for 3 days. Moreover, all substances administered upregulate ALP gene expression to a lesser extent when HPCs are stimulated with LPS. More specifically, in the presence of 2 mM or 5 mM NAC, the relative expression of ALP is 12.9- and 7.6-fold greater than the untreated sample after 3 days, while in the presence of LPS the fold increases are lower. Likewise, BSO upregulates ALP about 2.5-fold or 6.3-fold with or without LPS, respectively. In parallel, 5 or 25 µM fisetin in M2 enhance ALP expression after a 3 days’ exposure period, whereas ALP is hardly detectable with 50 µM fisetin. As expected, after 21 days in the presence of M2, the expression of ALP is downregulated in untreated HPCs. Yet, NAC slightly but significantly enhances ALP expression mainly in the presence of LPS. Then, ALP expression is still significantly enhanced with 25 or 50 µM fisetin independently from LPS.

### 3.6. IL-6 Secretion

**Figure 6:**
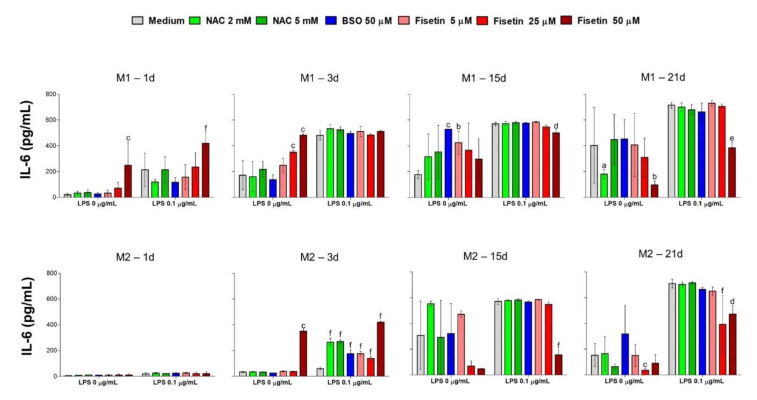
Interleukin-6 released from human pulp cells in the various experimental conditions after 1, 3, 15 and 21 days. Bars represent mean values ± standard deviations combined from duplicates in two independent experiments (n = 4). M1 = complete growth medium; M2 = complete growth medium with dexamethasone and β-glycerophosphate. a = *p* < 0.01, b = *p* < 0.001 and c = *p* < 0.0001 between 0 µM and treated samples without LPS (0 µg/mL); d = *p* < 0.01, e = *p* < 0.001 and f = *p* < 0.0001 between LPS alone and treated samples in the presence of LPS (0.1 µg/mL). IL-6 release is earlier amplified in LPS-stimulated HPC under non-odontogenic conditions than under odontogenic ones.

Secretion of IL-6 is upregulated by LPS during the immunomodulation, and the expression of this cytokine has been recently related to MSC commitment to osteogenesis. Therefore, a time course of released IL-6—which is has not yet been examined in this population of HPCs—was run out and its amount was quantified in the various experimental conditions every 72 h, starting from 1 day to 21 days of culture (Figure 6 and Figure S5). LPS stimulation amplifies IL-6 secretion from HPCs under all experimental conditions and after all times of exposure. Interestingly, after 1 day of culture, IL-6 is hardly detectable when cells are cultivated in M2 medium, even in the presence of LPS. On the contrary, cytokine secretion in M1 is dramatically increased by LPS compared to untreated cells (213.2 pg/mL and 20.8 pg/mL, respectively). Moreover, all substances administered modulate IL-6, mainly fisetin at the concentration of 50 µM. Likewise, after 3 days of exposure in M1, the release of IL-6 is enormously raised by LPS to 481 pg/mL independent from other substances. In contrast, a significant dose-dependent increase in IL-6 levels is induced by fisetin in the absence of LPS (471 pg/mL with fisetin 50 µM). In the presence of M2 after 3 days, only the highest dose of fisetin caused an increase in IL-6 secretion in HPCs without LPS. In contrast to the stimulation in M1, LPS fails in increasing IL-6 when HPCs are cultivated in the M2 medium. In parallel, all substances modulate cytokine secretion, mainly NAC and fisetin. After 15 days, LPS stimulation increases the IL-6 amounts both in M1 and M2 to around 600 pg/mL. IL-6 is highly produced in the presence of all substances without LPS, mainly with BSO (528 pg/mL) and 5 µM fisetin in M1 (424 pg/mL). In the presence of LPS, the decreased secretion of IL-6 seems to be independent from the substances both in the M1 and in the M2, except for 50 µM fisetin M2. Finally, after 21 days of LPS stimulation, the concentration of IL-6 remains high at around 700 pg/mL both in M1 and M2 (around 700 pg/mL), and the modulation in the presence of LPS is still independent from substances except for 25 and 50 µM fisetin which decrease the amount of IL-6 to about 400 pg/mL in M2.

## 4. Discussion

Being functionally responsible for the maintenance and repair of tissues of the dentin-pulp complex and its associated immune system, pulp cells own a high regenerative capacity and respond to various types of damage. Consequently, dental MSCs proliferate and migrate into the damaged tissue, differentiate into odontoblast-like cells, and form reparative dentin as the main mechanism leading to reparative dentinogenesis [23]. LPS is a glycolipid and a pathogenic molecule of Gram-negative cariogenic bacteria capable of inducing dental pulp inflammation. The endotoxin binds to its Toll-like receptor (TLR)-4 and triggers the activation of the redox-sensitive transcription factor nuclear factor kappa-light-chain-enhancer of activated B cells (NFκB) or MAPKs, thus resulting in the release of pro- or anti-inflammatory cytokines, such as IL-6 [24].

In this study, we investigated the relevance of the cellular redox balance of vital dental pulp cells functions. To this end, pulp cells were stimulated with a low concentration of LPS (0.1 µg/mL) to induce a perturbation of the intracellular redox equilibrium and mimic inflammation. In addition, cells were exposed to BSO as an irreversible inhibitor of γ-glutamylcysteine synthetase (γ-GCS) to exacerbate the intracellular oxidative stress [18], and to the well-known reducing agent NAC, capable of successfully counteracting oxidation in several cell models [18,25]. Moreover, the natural flavonol fisetin was administered to HPCs. Fisetin (3,3′,4′,7-tetrahydroxyflavone) is a dietary flavonoid found in various fruits and vegetables and has shown strong anti-inflammatory, antioxidant, anti-tumorigenic, anti-invasive, anti-angiogenic, anti-diabetic, neuroprotective, and cardioprotective effects in cell cultures and in animal models relevant to human diseases [19]. However, its mechanism of action is still under investigation and its potential role in the mineralization process has been only recently disclosed [26]. Preliminary analyses on metabolic activity and the generation of ROS were performed to establish the accuracy of our experimental model (Figure 1, Figure 2 and Figure S2). Only the highest concentrations of NAC decrease cell metabolic activity mainly after 3 days; whereas, in the presence of fisetin, a bell-shaped trend is registered, and this flavonoid starts to be cytotoxic at concentrations higher than 50 µM. This could be ascribed to the so-called antioxidant-paradox, broadly discussed elsewhere [27]. In light of this, concentrations of fisetin tested non-cytotoxic under the current experimental conditions were used for further analyses in the present investigation. Moreover, considering that cell metabolic activities towards increasing concentrations of the various substances were comparable in the M2 and M3 media, and that ascorbic acid contained in the M3 medium could influence the redox homeostasis of cells reacting with radical compounds [28], only the M1 and the M2 media were used for further analyses. ROS measurements were instead performed only in complete medium M1 to detect the influence of the single substances also in combination with LPS, avoiding any other interference from additives used in the odontogenic media M2 or M3. As expected, BSO increases the intracellular concentration of reactive oxygen species measured by DCF already at the earliest exposure time (3 h), while the two antioxidants exert their proper activity reducing them in a dose-dependent manner over the time of the experiment. Thus, these suitable experimental conditions were used to analyze the relevance of parameters related to mineralization and odontoblastic differentiation. 

Since the present paper aims to investigate the relationship underlying oxidative stress and mineralization occurrence, evaluating the modulation of CD markers involved in the HPC odontogenic commitment under pro- and antioxidant conditions is crucial. A minimal phenotypic pattern for the identification of MSCs requires them to be positive for CD90, CD73 and CD105 expression, while being negative for CD34 or CD45 [8]. As for CD140a (platelet-derived growth factor receptor A, PDGFRα), it is reported to identify a population of progenitor cells of neural crest origin and it has been included in this investigation due to its weak expression found in cells previously isolated from the dental pulp-interface and immunophenotypically characterized [20]. The positivity of MSCs from the dental pulp to the various cell markers is differentially modulated under the odontogenic commitment [17]. Firstly, CD90 (Thy-1) is found decreased in a time-dependent manner, meaning that cell stemness is reduced towards the osteogenic differentiation [17]. Secondly, the ecto-5′-nucleotidase CD73 is upregulated under osteogenic conditions and highly expressed in odontoblasts, as it is involved in adenosine production and in the enhancement of bone metabolism through the Wntβ-catenin signaling [29]. Thirdly, expression levels of the co-receptor for ligands of the transforming growth factor (TGF)-β superfamily (endoglin or CD105), have been found to increase rapidly in freshly harvested MSCs after 4–7 days of culture, and afterwards they gradually decrease during differentiation [30]. Regarding our experimental model, we show that the stimulation with a low LPS concentration does not affect the immunophenotypic profile of HPCs compared to controls. This is in accordance with the heavily calcium deposition shown with the Alizarin red staining at 21 days in M2. Similar findings were previously reported [31]. This intriguing observation can be plausibly related to a positive role of low concentration of nitric oxide (NO) in the stimulation of odontogenic differentiation [32], because LPS is an enhancer of NO release through the activation of NFκB and the subsequent induction of nitric oxide synthase (iNOS) [33]. On the other hand, a massive NFκB activation consequent to an exaggerated NO production has been reported to impair differentiation of MSCs by promoting β-catenin degradation [34]. Thus, pathways underlying the promotion of HPC odontogenic differentiation under sub-toxic LPS-stimulation should be further investigated, along with the potential control of mineralization not only through oxygen radicals but also through the modulation of nitrosative species.

In parallel, in the presence of BSO, a decrease in CD73 and CD140a is registered, as well as a lack of mineralization. It is plausible to assume that the administration of BSO, causing a burst of oxidative stress, leads to cytotoxicity and does not allow for the odontogenic commitment of HPCs. Moreover, BSO, depleting the GSH pool and thus presumably impairing the GSH/GSSG ratio, can affect osteogenic activity as reported elsewhere [35]. Next, the presence of NAC affects the enhanced expression of CD73 found in controls mainly at the highest concentration. In parallel, mineralization is decreased with 5 mM NAC in the M2 medium, especially in the absence of LPS. NAC has been widely described as a direct ROS scavenger [36] and it has been demonstrated that the MSC osteogenic commitment is under redox control with low amounts of oxygen radicals acting as molecular signals [14,17]. The negative effect on the expression of CD markers and even more on the mineralized matrix deposition can be observed with fisetin in a dose-dependent manner, in accordance with the recent work of Lorthongpanich and colleagues [26]. This is extremely evident when one considers the decrease in positivity of cell populations to CD73 and CD140a (Figure 3c,d, respectively) and the lack of Alizarin red staining when cells are exposed to 25 and 50 µM fisetin independently from the presence of LPS. Moreover, the microscopic observation of treated cell cultures reveals a fisetin-dependent decrease in cell density and attachment, which are crucial in triggering mineralization. Notably, this observation lays the grounds for further investigations regarding the role of flavonoids in the mineralization process. Indeed, a low dose-fisetin administration could be beneficial, if ROS is properly decreased and the calcium deposition allowed. It is currently believed that a regulated and moderated ROS production promotes essential signaling pathways such as Erk1/2 phosphorylation, which modulates cell functions such as differentiation. Moreover, numerous reports describe the importance of a redox control of MSC differentiation [37]. Furthermore, fisetin and other polyphenolic structures that own a catechol ring are particularly prone to specific aromatic electron delocalization which may happen as a result of contact with hydrogen acceptors, quinone, and vicinal diketones [38]. In addition, multihydroxyflavonols can undergo several metabolic reactions faster than other substances in a large plethora of culture media producing metabolites in different amounts whose effects on cell viability are not clarified [39]. Again, the administration of exogenous antioxidants can be a double-edged sword in the cellular redox state, due to a subtle equilibrium existing between beneficial effects at physiologic doses versus deleterious effects at high doses [40].

Bone morphogenic protein 2 (BMP2) is widely known as a potent growth factor early expressed in odontogenic precursors. To differentiate MSCs, BMP-2 binds to type I and type II serine/threonine kinase receptors on target cells, activating Smad (canonical) and non-Smad (non-canonical) signaling pathways, which ultimately activates odontogenic genes such as Runt-related transcription factor 2 (RUNX2) and osterix (Osx) [41]. In parallel, alkaline phosphatase (ALP) is highly expressed in cells of mineralized tissue and is critical in the formation of hard tissue. ALP functions as an inorganic phosphate rate enhancer and facilitates mineralization as well as reducing the concentration of extracellular pyrophosphate, an inhibitor of mineral formation [42]. Surprisingly, gene expression levels of BMP2 in our experimental model are clearly enhanced in samples treated with substances which do not allow for the achievement of mineralization after 21 days, for instance, 5 mM NAC (21 days in M2), 25 µM fisetin (after 21 days) and 50 µM fisetin (in all the experimental conditions, except for 21 days M2). It is plausible to assume that the observed gene upregulation is a positive feedback mechanism in a continuous effort of HPCs to achieve hard tissue formation. Simultaneously, upregulation of the ALP gene is registered for NAC at 21 days only in M1, which plausibly does not lead to a functional enhanced activity of the enzyme due to the decreased mineralization observed at the same experimental time. In parallel, ALP expression is not enhanced in the M2 conditions. The optimal pH for the ALP activity ranged between 8.5 and 9.5 [43]. In light of this, it can be speculated that, as for BMP2, the ALP gene is continuously up regulated but the activity of the enzyme is not optimal, due to the acidity of the environment caused by the high concentration of NAC. This is not observed in the M2 medium, resulting indeed in a better deposition of calcium nodules. As for fisetin, a strong induction is registered after 21 days in the M1 medium, and a weak one can be observed also in the presence of M2. Once again, although the expression of the ALP gene is triggered, there is a failure in its functional activity due to the lack of mineralized matrix deposition.

The term osteoimmunology refers to the existing link between the immune and the skeletal system. Pro-inflammatory cytokines, such as tumor necrosis factor (TNF)-α and IL-6 play important roles in immune responses and bone metabolism. LPS-induced secretion of TNF-α and IL-6 in macrophages is well documented, while IL-6 was recently found to be a crucial mediator of the actions of osteoblasts and osteoclasts through sophisticated mechanisms, which reflect a dual effect of this cytokine [44]. It was shown that IL-6 contributes to bone remodeling in the early stages of fracture healing, and after fracture surgery, is involved in bone remodeling activation [4]. In the present data set, a significant induction of LPS-mediated IL-6 secretion can be observed in HPCs already after one day in the M1 medium, and it is maintained up to day 21, independently from other substance administered in parallel. Contrariwise, cells grown in the M2 medium strongly secrete IL-6 in the presence of LPS only after 15 days of culture. This anti-inflammatory effect could be caused by the presence of the low concentration of dexamethasone in the odontogenic medium M2 [45]. Moreover, it could be speculated that in the M1 conditions, an early IL-6 secretion has a canonical inflammatory function, being involved in the recruitment of activated immune cells in the oral mucosa, which in turn secrete IL-17 contributing to the eradication of oral bacteria [46]. The latest secretion of IL-6 from HPCs grown in the osteogenic medium M2, could be related to a cytokine-mediated control of cell odontogenic commitment, as already reported in the presence of bone grafts for regenerative purposes. In this experimental model, IL-6 increased in a time-dependent manner started from day 15 in parallel with the activation of antioxidant proteins such as nuclear factor erythroid 2–related factor 2 (Nrf2), phosphorylated extracellular signal-regulated kinase (Erk 1/2) and catalase [15,47], thus indicating a redox-control of the odontogenic commitment through IL-6. In addition to Nrf2-related antioxidant enzymes, a spontaneous upregulation of silent information regulator type 1 (SIRT1) and glutathione peroxidase 1 has been reported during MSC osteogenic commitment, which accounted for the enhanced resistance to oxidative stress upon osteogenic differentiation without the administration of antioxidant supplementations [48].

## 5. Conclusions

In the present work, pro- and antioxidant compounds with different mechanisms of action were used to imbalance the redox equilibrium and to provide experimental evidence for a relationship between oxidative stress and vital functions of MSCs from the dental pulp. Intriguingly, our data demonstrate that HPCs do not fail to mineralize after stimulation with a low dose of LPS in odontogenic conditions. This observation lays the groundwork for further investigations on the molecular mechanisms underlying this process in HPCs. Redox homeostasis in HPCs is disturbed by pro- and antioxidant compounds. The administration of a pro-oxidant as well as physiologically relevant doses of antioxidants, independently from their mechanism of action, discloses a paradoxical effect on the mineralization occurrence, as HPCs are not capable of depositing calcium in the extracellular matrix. The expression of CD markers related to odontogenic commitment was influenced accordingly. Finally, the secretion of IL-6 was differentially modified depending on a variety of factors such as LPS, pro- or antioxidants, culture media and exposure periods.

In summary, the present study provides experimental evidence that critical functions of HPCs causally depend on a tightly regulated cellular redox balance. Therefore, these data are a valuable basis for a more detailed analysis of a relation between IL-6 release and mineralization. This potential dual role of IL-6 could be addressed, for instance, by investigating the differentiation of HPCs after the neutralization of IL-6 in a subsequent investigation completed with a detailed statistical analysis of the correlation between ROS production, expression of differentiation markers and IL-6 formation.

## Data Availability

Data is contained within the article and Supplementary Materials.

## Supplementary Materials

The following supporting information can be downloaded at: https://www.mdpi.com/article/10.3390/antiox11010023/s1, Figure S1: Cell metabolic activity of human pulp cells in the presence of the different media. The M1 is set as 100%. Bars show mean values ± standard deviations summarized from individual values independent experiments (n = 4). M1 = complete growth medium; M2 = complete growth medium with dexamethasone and β-glycerophosphate; M3 = complete growth medium with dexamethasone, β-glycerophosphate and ascorbic acid; Figure S2: Cell metabolic activity in human pulp cells. Cell cultures were exposed to LPS, NAC, BSO or fisetin for 72 h. Optical density readings in untreated cultures (0 µM) were set to 100%. Bars show mean values ± standard deviations summarized from individual values in independent experiments (n = 4). M1 = complete growth medium; M2 = complete growth medium with dexamethasone and β-glycerophosphate; M3 = complete growth medium with dexamethasone, β-glycerophosphate and ascorbic acid. Lower case letters indicate significant differences between untreated cell cultures (0 µM) and treated samples: a (*p* < 0.01), b (*p* < 0.001) and c (*p* < 0.0001); Figure S3: Immunophenotypic profile of human pulp cells. Cell cultures were treated under various experimental conditions for 1 day. Mean fluorescence intensity ratios (MFI ratio) for surface markers CD90, CD73, CD105, and CD140a with M1 and M2 were obtained as described in Materials and Methods. Bars represent the MFI of the sample divided by the fluorescence intensity of related isotype controls as mean values ± standard deviations summarized from individual samples in two independent experiments. M1 = complete growth medium; M2 = complete growth medium with dexamethasone and β-glycerophosphate. a = *p* < 0.01, b = *p* < 0.001 and c = *p* < 0.0001 between 0 µM and treated samples without LPS (0 µg/mL); d = *p* < 0.01, e = *p* < 0.001 and f = *p* < 0.0001 between LPS alone and treated samples in the presence of LPS (0.1 µg/mL); Figure S4: Microscopic analysis of cell culture treated with fisetin with or without LPS in the presence of M1 after 3 days. (A) Untreated control. (B-C-D) Fisetin 5-25-50 µM, magnification 100× (E) Fisetin 50 µM, magnification 200× (F) LPS 0.1 µg/mL. (G-H-I) Fisetin 5-25-50 µM with LPS, magnification 100× (L) Fisetin 50 µM with LPS, magnification 200×; Figure S5: Interleukin-6 released from human pulp cells in the various experimental conditions after 6, 9, 12 and 18 days. Bars represent mean values ± standard deviations combined from duplicates in two independent experiments (n = 4). M1 = complete growth medium; M2 = complete growth medium with dexamethasone and β-glycerophosphate. a = *p* < 0.01, b = *p* < 0.001 and c = *p* < 0.0001 between 0 µM and treated samples without LPS (0 µg/mL); d = *p* < 0.01, e = *p* < 0.001 and f = *p* < 0.0001 between LPS alone and treated samples in the presence of LPS (0.1 µg/mL).
