# Supplementary material for: Relevance of Cellular Redox Homeostasis for Vital Functions of Human Dental Pulp Cells
Source: Antioxidants (Basel). 2021 Dec 23;11(1):23. doi: 10.3390/antiox11010023 (PMC8772760; doi:10.3390/antiox11010023)
Supplement: Supplementary file 1 [file antioxidants-11-00023-s001.zip › antioxidants-1491536-supplementary.pdf]

Article

Relevance of Cellular Redox Homeostasis for Vital Functions of Human Dental Pulp Cells

Marialucia Gallorini [1,2,*], Matthias Widbiller [2], Carola Bolay [2], Simone Carradori [1,*], Wolfgang Buchalla [2], Amelia Cataldi [1] and Helmut Schweikl [2]

[1] Department of Pharmacy, University "G. d'Annunzio" Chieti-Pescara, Via dei Vestini 31, 66100 Chieti, Italy; cataldi@unich.it

[2] Department of Conservative Dentistry and Periodontology, University Hospital Regensburg, 93042 Regensburg, Germany; matthias.widbiller@ukr.de (M.W.); carola.bolay@ukr.de (C.B.); wolfgang.buchalla@ukr.de (W.B.); helmut.schweikl@ukr.de (H.S.)

* Correspondence: marialucia.gallorini@unich.it (M.G.); simone.carradori@unich.it (S.C.)

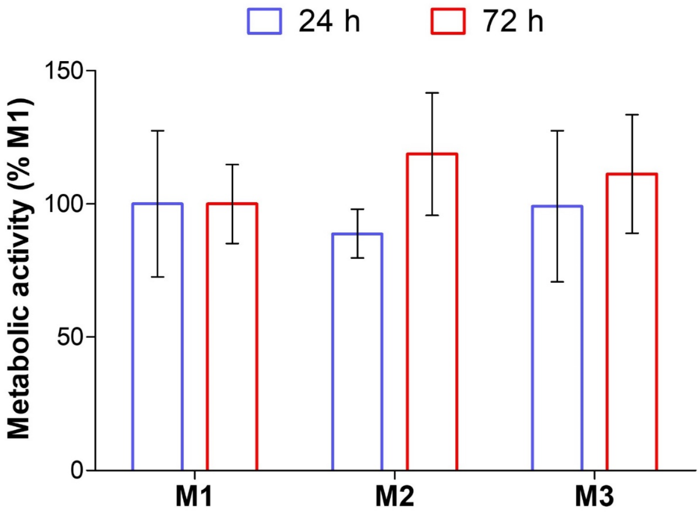

Figure S1. Cell metabolic activity of human pulp cells in the presence of the different media. The M1 is set as 100%. Bars show mean values ± standard deviations summarized from individual values independent experiments (n = 4). M1 = complete growth medium; M2 = complete growth medium with dexamethasone and β-glycerophosphate; M3 = complete growth medium with dexamethasone, β-glycerophosphate and ascorbic acid.

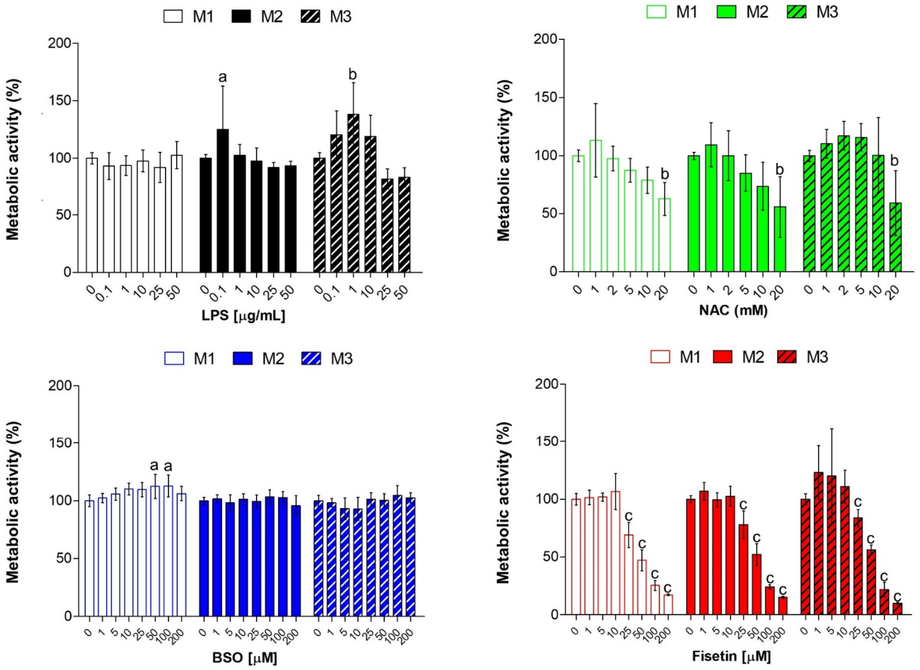

Figure S2. Cell metabolic activity in human pulp cells. Cell cultures were exposed to LPS, NAC, BSO or fisetin for 72 h. Optical density readings in untreated cultures (0 µM) were set to 100%. Bars show mean values ± standard deviations summarized from individual values in independent experiments (n = 4). M1 = complete growth medium; M2 = complete growth medium with dexamethasone and β-glycerophosphate; M3 = complete growth medium with dexamethasone, β-glycerophosphate and ascorbic acid. Lower case letters indicate significant differences between untreated cell cultures (0 µM) and treated samples: a (p<0.01), b (p<0.001) and c (p<0.0001).

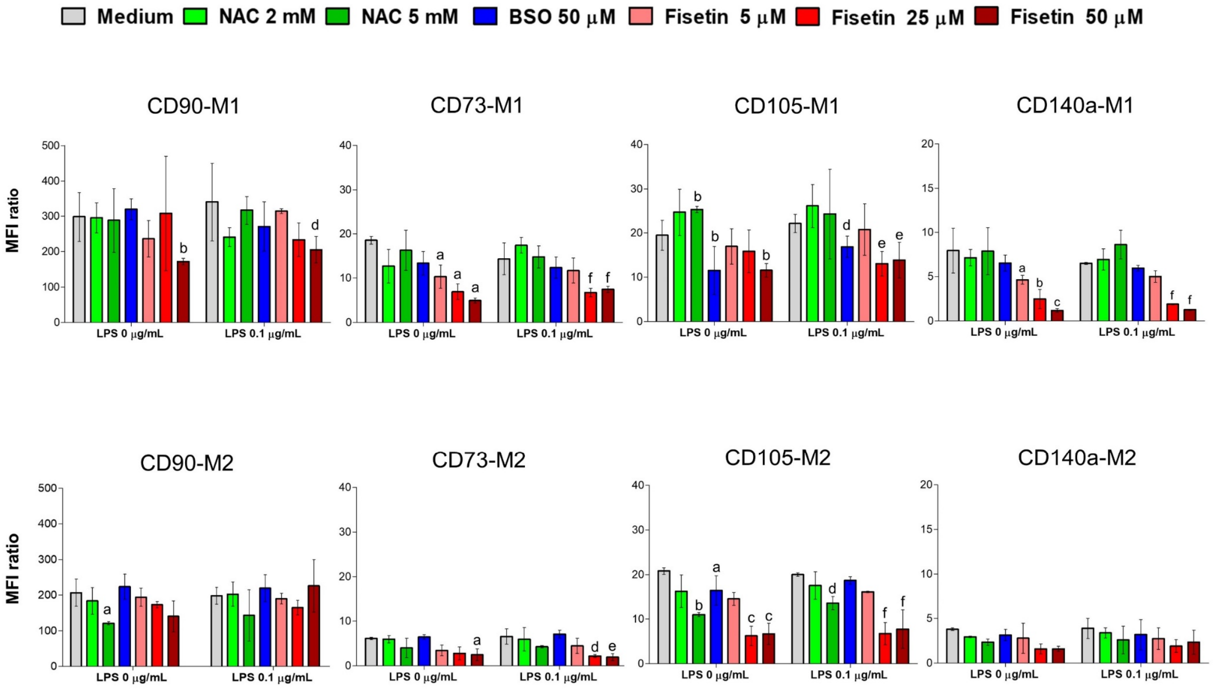

Figure S3. Immunophenotypic profile of human pulp cells. Cell cultures were treated under various experimental conditions for 1 day. Mean fluorescence intensity ratios (MFI ratio) for surface markers CD90, CD73, CD105, and CD140a with M1 and M2 were obtained as described in Materials and Methods. Bars represent the MFI of the sample divided by the fluorescence intensity of related

isotype controls as mean values ± standard deviations summarized from individual samples in two independent experiments. M1 = complete growth medium; M2 = complete growth medium with dexamethasone and β-glycerophosphate. a = p<0.01, b = p<0.001 and c = p<0.0001 between 0 μM and treated samples without LPS (0 μg/mL); d = p<0.01, e = p<0.001 and f = p<0.0001 between LPS alone and treated samples in the presence of LPS (0.1 μg/mL).

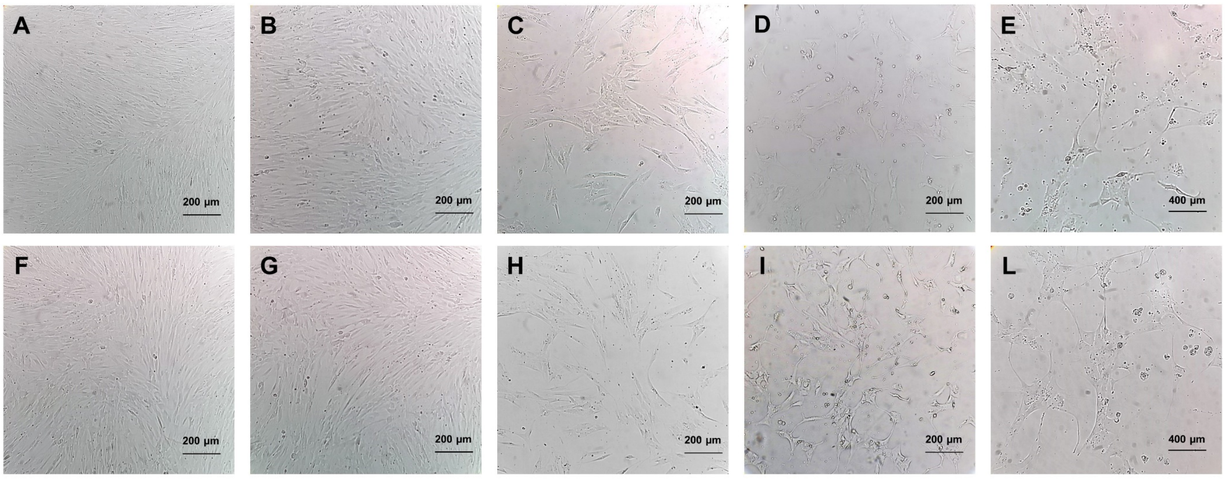

Figure S4. Microscopic analysis of cell culture treated with fisetin with or without LPS in the presence of M1 after 3 days. (**A**) Untreated control. (**B**–**D**) Fisetin 5-25-50 μM, magnification 100 x (**E**) Fisetin 50 μM, magnification 200 x. (**F**) LPS 0.1 μg/mL. (**G**–**I**) Fisetin 5-25-50 μM with LPS, magnification 100 x. (**L**) Fisetin 50 μM with LPS, magnification 200 x.

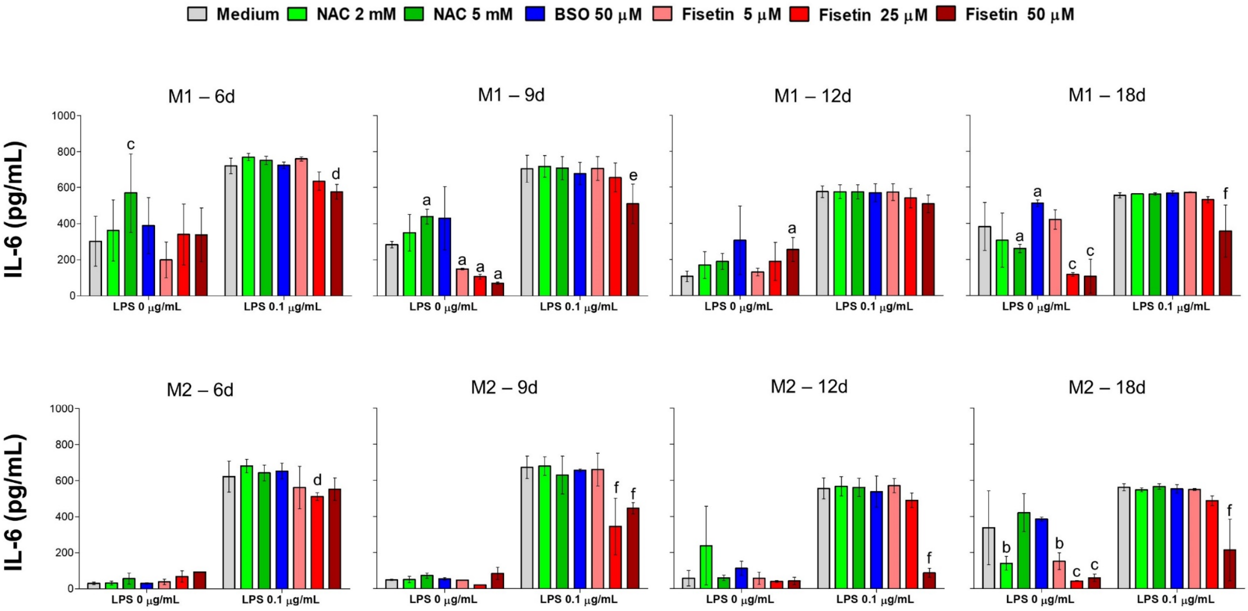

Figure S5. Interleukin-6 released from human pulp cells in the various experimental conditions after 6, 9, 12 and 18 days. Bars represent mean values ± standard deviations combined from duplicates in two independent experiments (n = 4). M1 = complete growth medium; M2 = complete growth medium with dexamethasone and β-glycerophosphate. a = $p < 0.01$, b = $p < 0.001$ and c = $p < 0.0001$ between 0 μM and treated samples without LPS (0 μg/mL); d = $p < 0.01$, e = $p < 0.001$ and f = $p < 0.0001$ between LPS alone and treated samples in the presence of LPS (0.1 μg/mL).
